# Bone marrow mesenchymal stem cells participate in prostate carcinogenesis and promote growth of prostate cancer by cell fusion *in vivo*

**DOI:** 10.18632/oncotarget.9045

**Published:** 2016-04-27

**Authors:** Fei Luo, Tong Liu, Jianan Wang, Jian Li, Pengde Ma, Hao Ding, Guowei Feng, Dong Lin, Yong Xu, Kuo Yang

**Affiliations:** ^1^ Department of Urology, The Second Hospital of Tianjin Medical University, Tianjin Institute of Urology, Tianjin, China; ^2^ Department of Urology, Tianjin Union Medical Center, Tianjin, China; ^3^ Department of Genitourinary Oncology, Tianjin Medical University Cancer Institute and Hospital, National Clinical Research Center for Cancer, Key Laboratory of Cancer Prevention and Therapy, Tianjin, China; ^4^ BC Cancer Research Centre, Vancouver, Canada

**Keywords:** bone marrow transplantation, prostate cancer, tumorigenesis, bone marrow-derived mesenchymal stem cells

## Abstract

The tumor microenvironment is comprised of diverse stromal cells that contribute towards tumor progression. As a result, there has been a growing interest in the role of bone marrow derived cells (BMDCs) in cancer progression. However, the role of BMDCs in prostate cancer (PCa) progression still remains unclear. In this study, we established GFP bone marrow transplanted TRAMP and MUN-induced prostate cancer models, in order to investigate the role of BMDCs in prostate cancer progression. By tracing GFP positive cells, we observed that BMDCS were recruited into mouse prostate tissues during tumorigenesis. GFP+/Sca-1+/CD45− BMDCs were significantly increased in the MNU-induced PCa group, as compared to the citrated-treated control group (2.67 ± 0.25% vs 0.67 ± 0.31%, *p* = 0.006). However, there were no significant differences found in GFP+/Sca-1+/CD45+ cell populations between the two groups (0.27 ± 0.15% vs 0.10 ± 0.10%, *p* = 0.334). Moreover, co-grafting of bone marrow mesenchymal stem cells (BMMSCs) and RM1 cells were found to promote RM1 tumor growth *in vivo*, and cell fusion was observed in RM-1+BMMSCs xenografts. Therefore, the data suggests that BMDCs can be recruited to the prostate during carcinogenesis, and that BMMSCs may promote the growth of PCa.

## INTRODUCTION

Prostate cancer (PCa) is the most commonly-diagnosed, non-skin cancer in men, and is also a major cause of cancer-related deaths [1–2]. Although significant improvements have been made in the diagnosis and treatment of PCa, most PCas will eventually progress into an androgen-independent, incurable stage, referred to as castration-resistant prostate cancer (CRPC) [3]. Therefore, a better understanding of the underlying molecular mechanisms during PCa tumorigenesis and progression, in addition to the identification of essential molecular targets, are urgently needed for both preventive and therapeutic purposes.

The interaction between the cancer and the stroma plays an important role in tumorigenesis and cancer progression. Bone marrow derived mesenchymal stem cells (BMMSCs) are a subset of bone marrow derived cells (BMDCs), and are capable of migration, self-renewal, participation in the repair of injured tissues [4], and differentiation into mesenchymal lineages (including osteoblasts, chondrocytes and adipocytes, under specific culture conditions [5–6]). BMMSCs have the ability to hone into sites of tissue injury, in order to restore homeostasis [7–8]. As has been previously documented, during lung contusions (alone or in combination with hemorrhagic shock), mobilized BMMDCs hone into the site of injury from the peripheral blood [9–10]. More importantly, it has been reported that cancer stem cells (CSCs) may derive from tumor-transformed BMMSCs, which play an important role in cancer development [11]. However, the role of BMMSCs and the extent of their contributions toward PCa development and progression are still unclear. Therefore, for this study we have established the TRAMP mice model of GFP bone marrow transplanted (BMT) and N-methyl nitrosourea (MNU) induced mice model of PCa in order to investigate the role of BMMSCs in prostate carcinogenesis.

## RESULTS

### Optimization of radiation dose for bone-marrow transplantation and establishment of GFP bone-marrow transplantation model

To determine the optimal radiation dose for bone-marrow transplantation (BMT), 20 mice were randomly divided into 4 groups and received 7.5, 8.5, 9.5 and 10.5 Gy of ^137^Cs irradiation. Within two hours after irradiation, each recipient mice was then injected with 2 × 10^6^ (100 μl) BMDCs, harvested from C57BL/6 donor mice. Most of the mice in the high-dose group (9.5, 10.5 Gy) were dead within 1 week following ^137^Cs irradiation. Bleeding, edema and necrosis of liver, lung, and gastrointestinal tissues were observed in these mice during follow-up autopsies (Figure 2C), while all the mice in the low-dose group (7.5, 8.5 Gy) survived (Figure 2B). There was found to be a significant difference in body weight loss, median survival time (MST), and white blood cell count of peripheral blood, between high-dose and low-dose groups (Table 1). White blood cell counts of the mice in the low-dose group was decreased and reached nadir approximately 1 week following irradiation. Then, after 2–3 weeks, the cell counts then return to normal levels. In view of this, 8.5 Gy was selected as the optimum ^137^Cs BMT radiation dose, at a rate of 0.708 Gy/min.

**Table 1:** Mouse body weight loss, white blood cells count, and survival percentage of each group (30 days limited)

Group	Weight loss (g)	Death	Survival (%)	MST (day)	WBC count (×10^9^)
7.5 Gy	3.20 ± 0.44	0	100	30	13.60 ± 2.50
8.5 Gy	4.12 ± 0.30	0	100	30	14.8 ± 1.33
9.5 Gy	4.56 ± 0.51	4	20	9 (4–30)	4.06 ± 4.98
10.5 Gy	5.02 ± 0.26	5	0	4 (3–7)	1.38 ± 0.29

GFP-positive donor bone marrow stem cells were harvested and transplanted in the manner as described in Materials and Methods. The GFP-positive cells in the peripheral blood of recipient mice were examined by flow cytometry, 4 weeks after the transplantation operation. The GFP positive rate of peripheral blood cells in the mice that received C57BL/6 bone marrow was less than 1%. By contrast, in the peripheral blood of the mice that received GFP positive bone marrow, more than 75% were observed to be GFP positive cells (Supplementary Figure S1). After 28 weeks, the population of GFP positive cells in the mice that received GFP positive bone marrow reached more than 90% (Supplementary Figure S2). This data suggest that the GFP-BMT mouse model is suitable for tracing BMDCs in recipient mice.

### Establishment of BMT TRAMP and MNU-induced model

**Figure 1:**
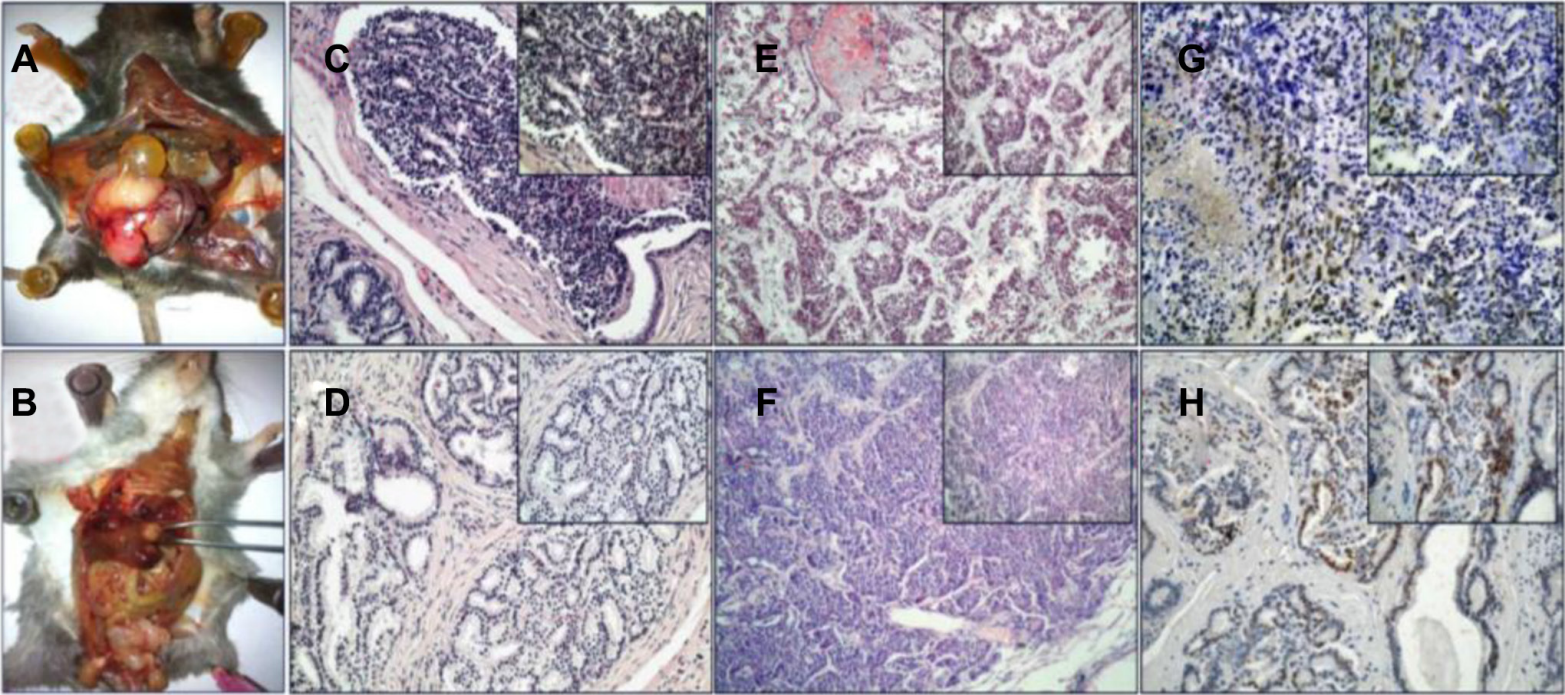
TRAMP transgenic mice (**A**, **B**) prostate tumor and metastasis tumor. (**C**, **D**) HE staining of prostate cancer. (**E**) Liver metastases of PCa. (**F**) Lung metastases of PCa. (**G**, **H**) IHC of SV40-Tag. (200×, 400×).

The contribution of BMDCs towards PCa tumorigenesis was studied in two PCa mouse models, i.e. TRAMP and MUN-induced model [12–14]. First, the SV-40 Tag was confirmed in TRAMP mice using PCR (Supplementary Figure S3). For a total of 20 TRAMP mice, PCa were observed in 55% (11/20) of the mice and 20% of the (4/20) mice showed tumor metastasis when they were sacrificed at an age of 36 weeks (Figure 1A, 1B). Local PCas demonstrated poor glandular formation, cellular atypia, and nuclear pleomorphism (Figure 1C, 1D). The PCa cells that invaded into liver or lung tissue displayed a more malignant phenotype (Figure 1E, 1F). We then undertook further investigation of the SV40-Tag in the tumor cells by using IHC staining. A markedly high epithelial SV40-Tag expression was then observed (Figure 1G, 1H). This data suggests that spontaneous PCa formation and metastasis can be consistently observed in TRAMP mice over time.

**Figure 2:**
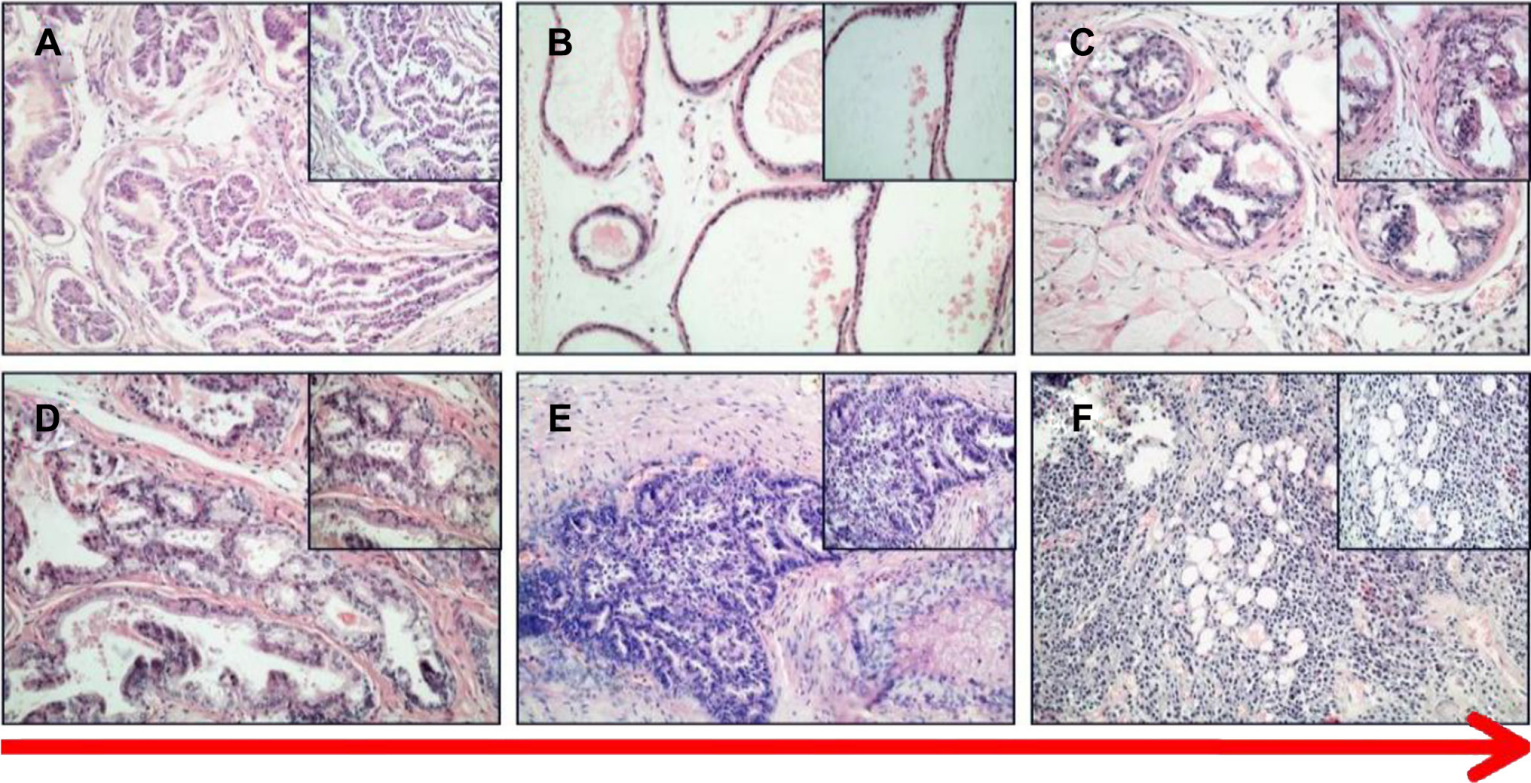
HE staining of PCa in MNU-induced mice (**A**) control. (**B**) prostate acinar hypertrophy. (**C**, **D**) PIN and primary cancer. (**E**, **F**) invasive cancer (200×, 400×).

To establish a MNU induced PCa model, MNU was injected into prostate tissue of 50 C57BL/6 mice (2 mg per mouse) every 3 weeks. A subsequent H&E stain showed that prostatic hyperplasia, PIN, and invasive PCa developed gradually during the process of MNU-injection. Similar to prostatic hyperplasia, cuboidal/columnar luminal cells, acinar lumen expansion, secretion retention, and a few interstitial components were observed in the MNU-induced mice at the end of the 2nd month (Figure 2B). Then, during the 4th month, induced prostatic intraepithelial neoplasia and primary PCas were observed. The epithelial cells of prostatic ducts proliferated to varying degrees, with enlarged nuclei and visible nucleoli. A complete or partial basal layer was observed (Figure 2C, 2D). Invasive prostate cancers were observed during the 5th–6th months of the MNU-induction, showing loss of basal layers of the prostate, a disordered gland structure, and obvious cell atypia (Figure 2E, 2F). As expected, the mice in the control group showed normal prostatic acinar structures (Figure 2A).

Hyperplasia, PIN, and primary PCa or invasive cancers were observed in 78% (39/50) of the MNU-treated mice, over time. No metastasis was identified in these mice (Table 2). This data suggested that MNU orthotopic injection, combined with testosterone, is able to induce PCa development in mice. The MNU-induced PCa model recapitulates the human tumor progression course, and therefore is an appropriate model for human PCa research.

**Table 2:** Tumor formation in control and MNU-induced mice

group	normal	hyperplasia	PIN	PCa	Invasive PCa
treatment	11	17	13	7	2
control	18	24	7	1	0

### BMDCs were recruited into prostate tissues during tumorigenesis

**Figure 3:**
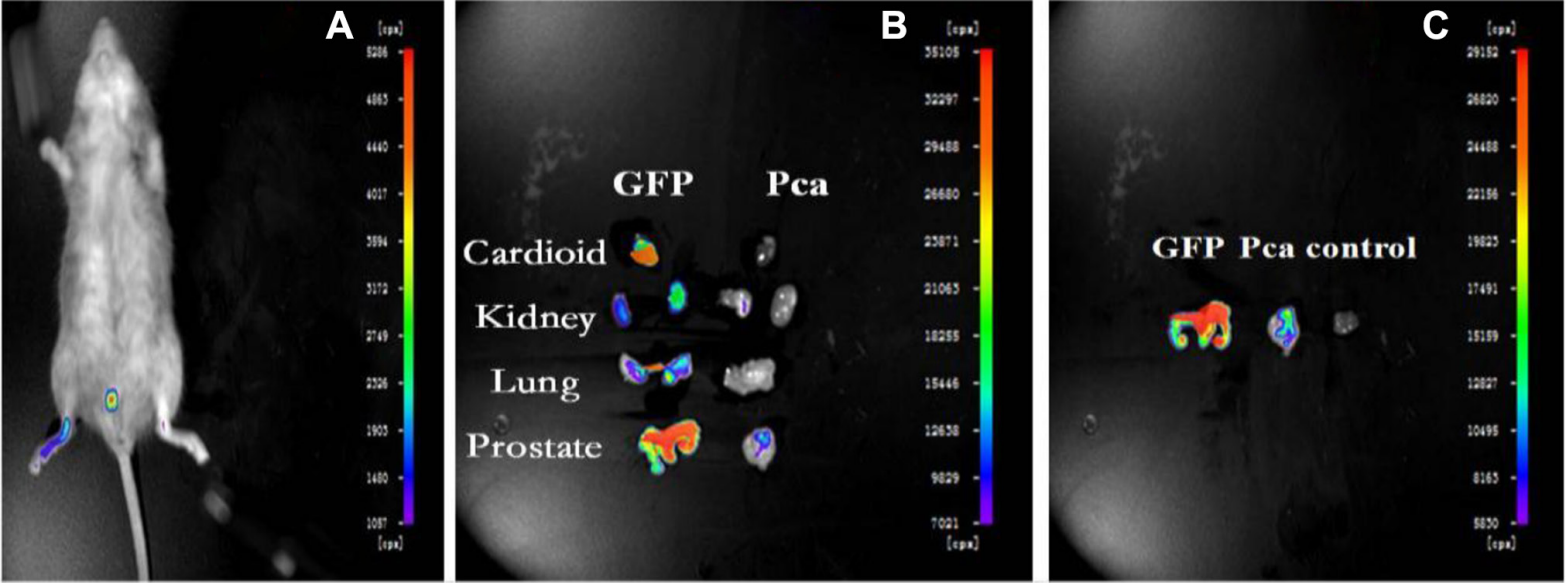
Small Living Animal Imaging Technology (**A**) GFP-BMT-PCa mice. (**B**) organs of chimeric and GFP mice. (**C**) prostate tissues in different mice.

In order to investigate the role of BMDCs during prostate tumorigeneisis, during the 4th month we examined the GFP-positive cells in the prostate cancer tissue of recipient mice by using a small living animal imaging system [15]. A weak positive signal was observed in the prostate of recipient mice, due to a blockage of signal by the skin and hair (Figure 3A). Thus, we further examined the GFP signal in multiple isolated organs after sacrificing the mice. A strong GFP-signal was detected in the prostate of GFP-BMT models, but not in the other organs of PCa models or the prostates of the wild type models. This result indicated that BMDCs were recruited into mouse prostate tissues during the tumorigenesis of prostate cancer (Figure 3B, 3C).

**Figure 4:**
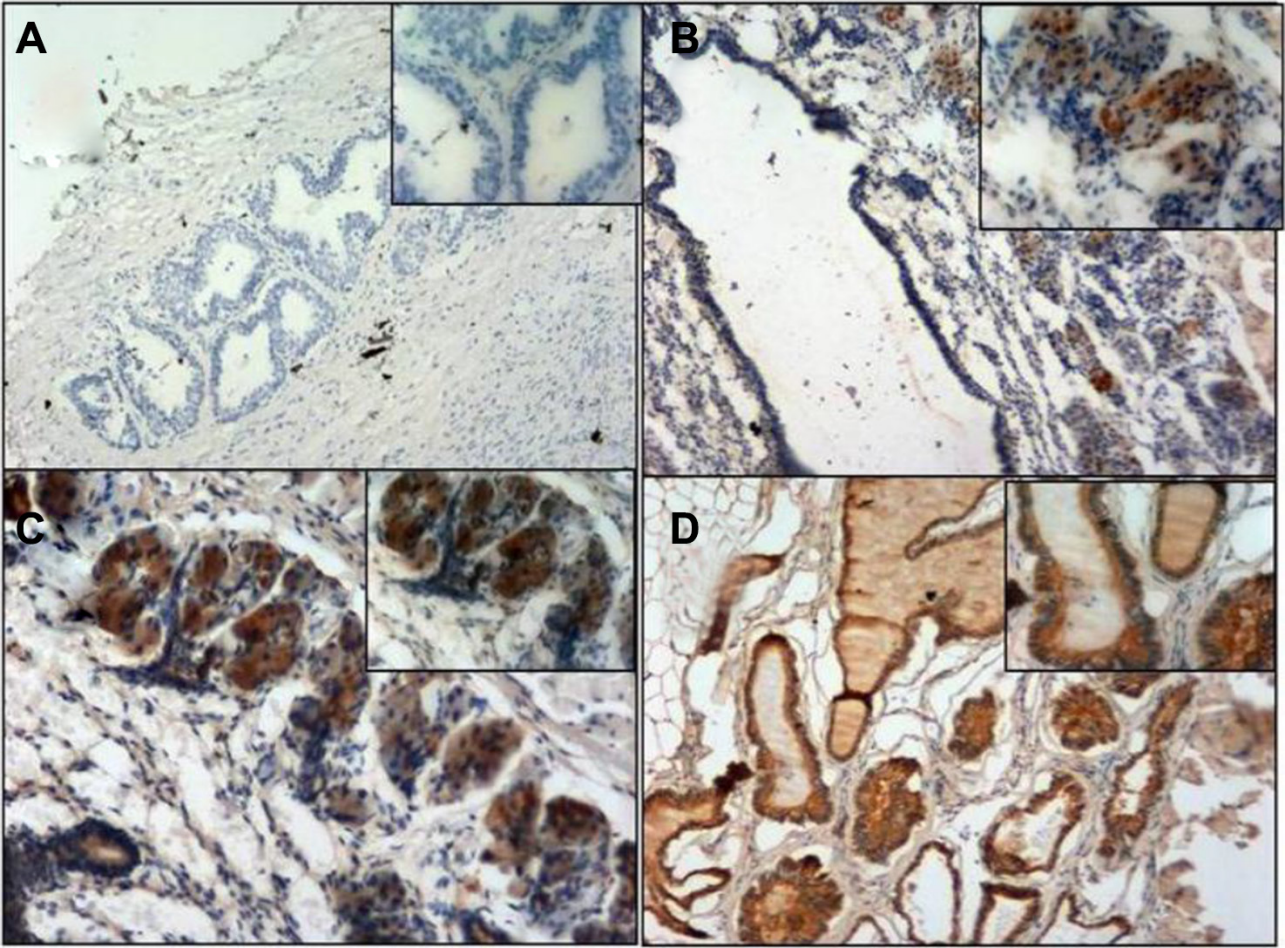
GFP immunohistochemistry staining (**A**) negative control. (**B**) Chimeric mice prostate. (**C**) Chimeric mice prostate. (**D**) GFP mice prostate (positive control). Magnification; A, B, D (100×, 200×) C: (200×, 400×).

To better clarify the role of BMDCs during tumorigenesis, GFP positive cells were further examined by immunohistochemistry staining in GFP-BMT mice during the 4th month. No GFP positive cells were detected in the wild type mice. In 27 out of 50 GFP-BMT mice, a significant increase in GFP positive cells was observed in the prostate, but not in any of the other organs (Figure 4B, 4C). These results suggest that BMDCs can infiltrate into the prostate and may be involved in the PCa progression of mice.

### Phenotype of BMDCs in PCa

**Figure 5:**
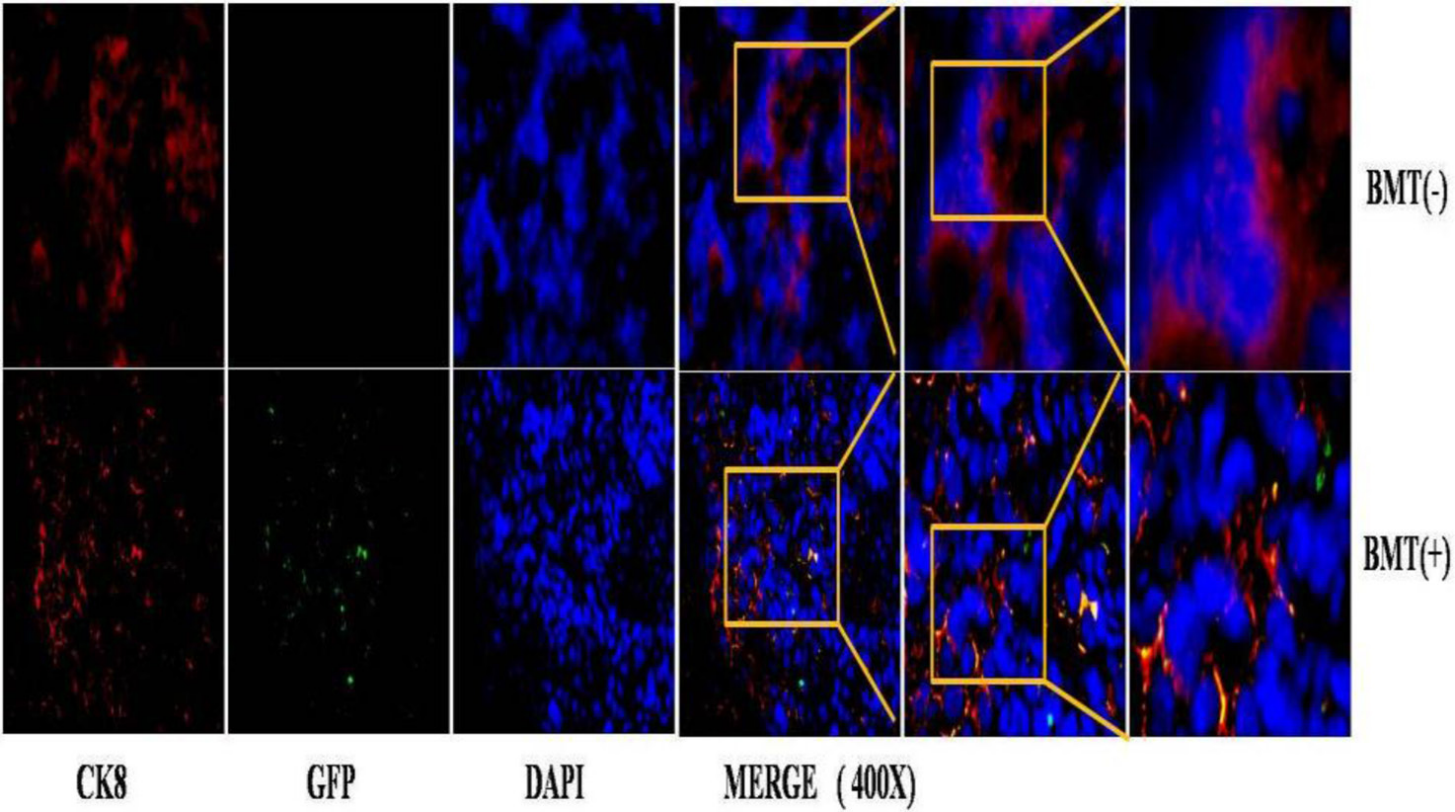
immunofluorescence staining of PCa in MNU-induced mice

To characterize the BMDCs in PCa, we examined the phenotype of GFP-positive BMDCs in BMT mice by immunofluorescence. The tissues were doubly stained with anti-GFP antibody in addition to either anti-CK8 or anti-SV40-Tag antibodies. Interestingly, a co-expression of GFP and CK8 was observed in the prostate epithelium of BMT-MNU induced mice (14/50 mice) (Figure 5). As the CK8 is a marker that is only expressed in epithelial cells [16], the co-expression of GFP and CK8 indicated that these cancer cells were derived from the bone marrow cell lineage, or otherwise developed by cell-fusion of BMDCs and prostate epithelial cells.

A similar phenomenon was observed in the BMT-TRAMP mice. The detection of GFP(+)/SV40-Tag(+) tumor cells indicated that cell fusion may be one of the mechanisms by which BMDCs promote tumorigenesis. A small population of GFP(+)/SV40-Tag(−) BMDCs was detected in the surrounding luminal cells, which suggests that different cell population of BMDCs are involved in inflammation. These results indicated that different subsets of BMDCs all hone to prostate tissue, and all have different roles during the development of PCa.

**Figure 6:**
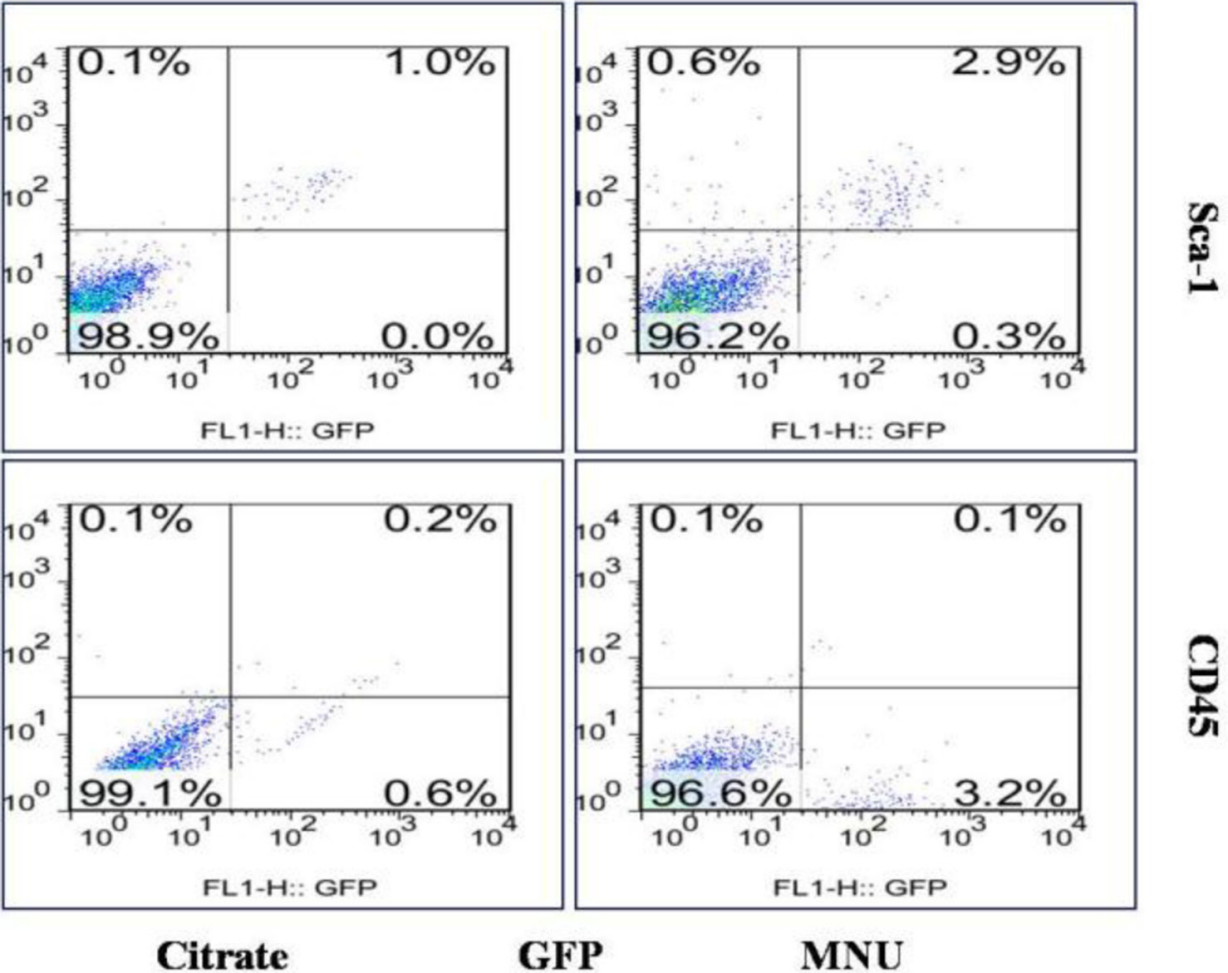
Flow cytometry of prostate cells Cells with the phenotype of GFP (+)/Sca-1 (+)/CD45 (−) were significantly increased in the MNU-induced mice.

There are two main types of stem cells in the BMDCs, i.e., hematopoietic stem cells (HSC) and mesenchymal stem cells (MSC). In order to further classify the identity of the BMDCs population that directly contributed towards tumorigenesis, CD45 (a pan-hematopoietic marker [17]) and Sca-1 (a well-recognized HSC marker [18]) were used to determine the phenotype of the cells infiltrating the PCa tissue. It was observed that GFP(+)/Sca-1(+)/CD45(−) BMDCs were significantly increased in the MNU-induced group, as compared to the citrated-treated control group (2.67 ± 0.25% vs 0.67 ± 0.31%, *p* = 0.006). There was no significant difference in the proportions of hematopoietic cells (GFP+/Sca-1+/CD45+) between the two groups (0.27 ± 0.15% vs 0.10 ± 0.10%, *p* = 0.334). It was also seen that more stem cells with the phenotype of GFP(+)/Sca-1(+)/CD45(−) were recruited into the lesion, in order to influence the progression of disease (Figure 6). These results suggest that BMMSCs may be involved in the tumorigenesis of prostate.

### BMMSCs promote PCa tumor growth by cell fusion *in vivo*

**Figure 7:**
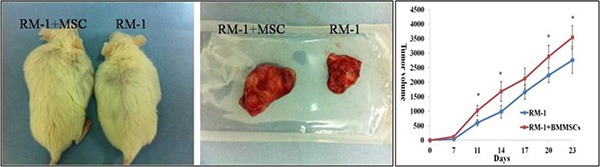
Tumor size and growth curve of RM-1 group and RM-1+BMMSCs group Asterisk indicates the time points that show a statistically significant difference in tumor volume.

To investigate the effects of BMMSCs on PCa tumor growth, RM-1 cells were injected into the SCID mice with and without the BMMSCs derived from GFP mice. The BMMSCs were harvested after CFU-F culture and tested by flow cytometry analysis (Supplementary Figure S4). Tumor volume was measured every 3 days, and a growth curve was drawn to record the changes in tumor volume. Interestingly, the tumor volume of the RM-1+BMMSCs group were found to be significantly higher than that of the RM-1 group at most time points (Figure 7).

**Figure 8:**
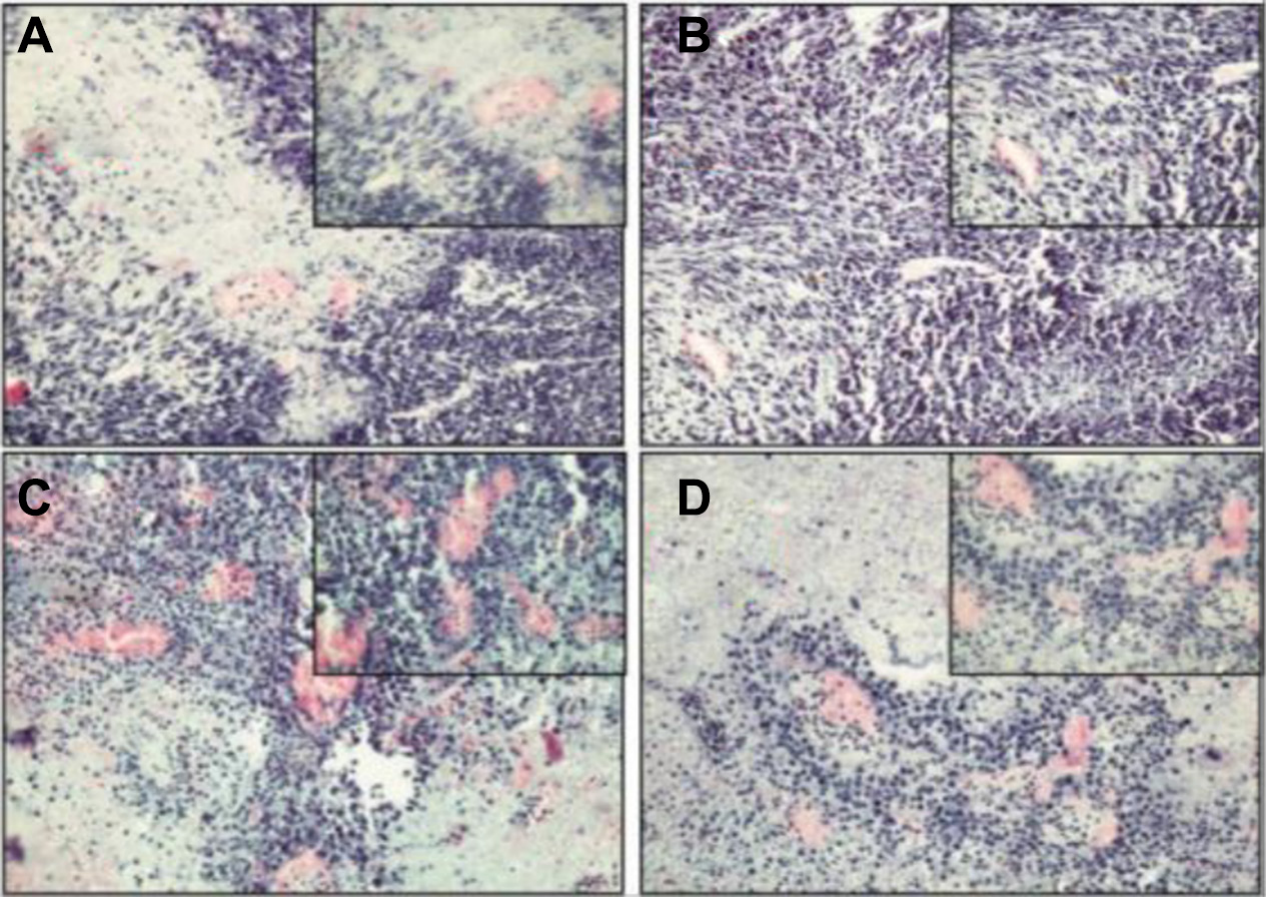
HE staining of xenograft tumor in the RM-1 group (A, B) and the RM-1+BMMSCs group (C, D)

H&E staining showed that the RM-1+BMMSCs group presented more vascular channels than the RM-1 group (Figure 8). In addition, GFP(+)/CK8(+) cells were observed in RM-1+BMMSCs xenografts, but not in the RM-1 group, therefore suggesting that cell fusion may play a role in PCa tumor growth. (Supplementary Figure S5).

## DISCUSSION

BMDCs have been shown to play an important role in tumor development. It has been reported that gastric cancer can originate from bone marrow-derived cells, in which BMMSCs have played a decisive role [10]. Similar results have also been observed in other types of cancer, suggesting that BMMSCs are closely associated with tumorigenesis. In addition, it has been reported that BMDCS participate during the course of androgen-modulated prostate regeneration [19]. In this study, in both TRAMP and MNU-induced PCa cancer models, we observed GFP-positive BMDCs in the prostate cancer tissue of the GFP-BMT recipient mice, thereby indicating that BMDCs can be recruited to the prostate and involved in PCa development.

We have also taken a deeper look into the composition of the BMDCs that are involved in PCa progression. Both CD45 (a pan-hematopoietic marker) and Sca-1 (a well-recognized HSC marker in mice) were used to identify the phenotype of the cells infiltrating PCa tissue. We observed an increase of GFP+/Sca-1+/CD45- cell populations in PCa tissues, as compared to the control group. In reference to the cell composition of BMDCs, we believe BMMSCs to be the main cells of recruitment [21, 22]. Our data suggests that BMMSCs were recruited into the PCa lesion and therefore influence the progression of disease.

To take a further look and functionally study the role of BMSCs in PCa progression, we co-injected GFP BMMSCs with RM-1 cells and monitored their influence on RM-1 cell growth. We observed a significant increase in tumor volume of the RM-1+BMMSCs group, compared to RM-1 group, which indicates that BMSCs can improve RM-1 tumor growth. Interestingly, H&E staining have also shown that the RM-1+BMMSCs group presents with more vascular channels. It is possible that the BMSCs have differentiated into the vascular endothelium, leading to an increase of vascularity and subsequently promoting the growth of the RM-1 xenograft. Moreover, GFP+/CK8+ cells were observed in RM-1+BMMSCs xenografts, suggesting that BMMSCs can fuse with prostatic epithelium. This result is consistent with studies in other types of cancer and implies that cell fusion may have a role in promoting cancer progression [20–23].

It has been previously reported that tumor progression is the consequence of an evolving interaction (by direct contact or regulation of growth factors) between tumor cells and tumor stroma cells [24–25]. Paracrine regulation of cytokines and chemokines were identified as key links in this process [26–28]. The increase of CSCs mediated by BMMSCs, via alteration of the CCL5-AR signaling pathway, can lead to an increased aggressiveness of PCa cells [29]. Both prostate tissue inflammation and blood circulation disorders can occur, as a result of cell injury by environmental factors. BMMSCs, as well as other stem cells, are attracted by a concentration gradient of chemokines (SDF-1) and selectively hone to the prostate in order to repair injury. Many types of immunomodulatory cells are involved in the process and secrete a wide variety of cytokines, leading to a special inflammatory microenvironment. Due to the interaction with the microenvironment, BMMSCs underwent a series of genetic recombination, secreting cytokines as a response. These cytokines then activated particular genes and pathways, in order to achieve reparation or induce apoptosis by cell fusion. Many researchers have previously demonstrated that BMMSCs can significantly contribute to the formation of carcinoma-associated fibroblasts and myofibroblast populations [30–31]. Once the inflammatory microenvironment has changed, the BMMSCs possessing the potential for differentiation, may present in a constant dedifferentiated state as a result of the inflammatory cytokines. During this period, oncogene activation and inactivation of tumor suppressor genes (or mitotic errors in BMMSCs) may lead directly to the generation of CSCs. Meanwhile, the inflammatory microenvironment promotes prostate carcinogenesis (Figure 8). In short, due to repeated cell damage and excessive proliferation-repair cycles in the chronic inflammatory microenvironment, BMMSCs undergo gene mutation, mitotic errors, and changes to the signal pathways of cell growth and differentiation, leading finally to malignant transformation.

In conclusion, BMDCs can migrate into the prostate and may be involved in prostate carcinogenesis. BMMSCs are the important cell populations involved in this progression and play a functional role in PCa development.

## MATERIALS AND METHODS

### Materials, animals and cell lines

Chemicals, solvents, and solutions were obtained from Sigma-Aldrich China Ltd (Shanghai, China), unless otherwise indicated. 4- to 6-week old wild type (WT) C57BL/6 mice, SCID mice, GFP and TRAMP transgenic mice were obtained from the Experimental Animal Center of the Academy of Military Medical Sciences. All the animals were maintained in a specific pathogen-free environment. All protocols are in compliance with the NIH Guideline for the Care and Use of Laboratory Animals and were approved by the Tianjin Medical University. RM1, a murine prostate cancer cell line purchased from Fuxiang Bio, Shanghai, was cultured in Dulbecco's modified Eagle's medium (DMEM) supplemented with 10% FBS.

### Bone-marrow transplantation (BMT)

Adult bone marrow was harvested from the femurs and tibias of donor GFP mice by flushing the bone marrow cavity using a 26-gauge needle. The bone-marrow-derived cells (BMDCs) were filtered through a 70-mm nylon mesh and layered over Histopaque-1077 (Sigma), centrifuged at 400–500 g for 3 min, washed, resuspended, and kept at 4°C for injection.

20 recipient C57BL/6 mice (6-weeks old) were divided into 4 groups and received lethal doses of 7.5, 8.5, 9.5, 10.5 Gy, respectively, of whole-body^137^Cs radiation. The optimal radiation dose was determined using the parameters of weight, white blood cells, and survival. Within two hours following irradiation, each recipient mouse was injected with 2 × 10^6^ (100 μl) BMDCs harvested from C57BL/6 donor mice.

**Figure 9:**
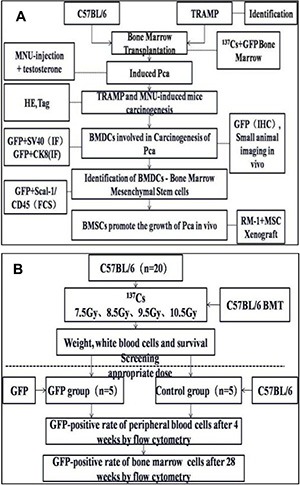
Experimental design (A) Wild type C57 mice and TRAMP mice were subjected to hematopoietic reconstitution from GFP(+) BMDCs PCa were induced in WT mice by MNU injection. Tumor formation was confirmed by checking HE or IHC staining of resected prostates. GFP-positive BMDCs were assessed by SAI and fluorescence microscopy. BMDCs involved in PCa tumorogenesis were isolated by flow cytometry. To observe the cancer-promoting role of bone marrow mesenchymal stem cells (BMMSCs), RM-1 xenografts with or without BMMSCs were grafted into mice, and then tumor formation was evaluated. (**B**) the choice of optimal radiation dose for BMT and the process of constructing the BMT model.

10 of the C57BL/6 mice were subjected to retrobulbar injection with about 2 × 10^6^ (100 μl) BMDCs from GFP transgenic or wild type mice. BMT efficiency was investigated by analyzing the GFP positive-rate of peripheral blood cells at 4 weeks, and of bone marrow cells at 28 weeks following transplantation in recipient mice, respectively (Figure 9B).

### Tumorigenesis study

50 6-week old WT C57BL/6 mice were castrated by orchiectomy. BM transplantation of GFP positive BMDCs were carried out at three weeks after castration. To induce prostate cancer in the recipient mice, an orthotopic injection of MNU (2 mg per mouse) was performed at 3 weeks after the BM transplantation. A negative control group was given orthotopic injections of citrate solution only (2 mg per mouse). The injection was performed at an interval of three weeks for 6 months, in combination with S.C. injection of testosterone daily (12.5 mg/kg·d-1). The mice were all euthanized at 27 weeks following BM transplantation. Prostates were promptly isolated for future analyses, as has been previously described.

In the investigate of tumor formation in MNU-induced mice, each group contained 50 mice. During the first 3 months, 3 mice were sacrificed every month from each group. During the 4th–5th months, 5 mice were sacrificed every month. At the end of the 6th month, all remaining mice were sacrificed.

### Immunohistochemistry and immunofluorescence

Formalin-fixed, paraffin-embedded murine prostate tissues were cut into serial 5-μm sections and mounted on glass slides. For histopathological analysis, routine hematoxylin and eosin (H&E) staining was carried out. After heat-induced epitope retrieval, immunohistochemical staining for GFP (Bioss Inc., Woburn, MA) and SV40 T-ag (Abcam Inc., Shanghai, China) were performed, following a previously described protocol.

For immunofluorescence staining, the prostate tissues were embedded into OCT compound and cut into serial 5-μm sections. The slides were stained with anti-CK8, anti-SV40-Tag, and anti-GFP primary antibodies. The nuclei were counterstained with DAPI.

The slides were photographed with a confocal laser-scanning microscope (Olympus).

### DNA extraction and Real-time polymerase chain reaction

Genomic DNA (total RNA) of TRAMP mice was extracted with Trizol reagent (Invitrogen), as according to the manufacturer's protocol. Real-time polymerase chain reaction (real-time PCR) was performed in a q-PCR detection kit (Promega Inc. Beijing, China). The primer sequences used are as follows: SV40-T Forward primer CAGAGCAGAATTGTGGAGTGG Reverse primer GGACAAACCACAACTAGAATGCAGTG (product size 474 bp) tcrd Forward primer CAAATGT TGCT TGT CTGGTG Reverse primer GTCAGTCGAG TGCACAGTTT (product size 200 bp).

### Flow cytometry analysis (FACS)

The isolated prostate tissues were rinsed with PBS buffer solution, cut into small pieces, and then immersed in collagenase IV for 1 hour in a 37°C water bath. The digested tissue was then filtered to obtain a single-cell suspension. Components of the cell population were analyzed by staining with APC labeled CD45 or Sca-1. Statistical Analysis.

All the data were presented as means ± SD and the 2-tailed *t* test was used to determine the differences between the two groups. Differences were considered statistically significant with *p* < 0.05.

### Study of tumour growth *in vivo*

The bone marrow of GFP mice was harvested from femurs and tibias by flushing the bone marrow cavity using a 26-gauge needle. The bone marrow cell suspension was filtered through a 70-mm nylon mesh and layered over Histopaque-1077 (Sigma), centrifuged at 1200 rpm for 5 min, washed, resuspended, and added 4 mL of 10% FBS complete medium. The cells were cultured in a 37°C, 5% CO2 incubator. On the third day, the cells that didn't stick to the plate wall were discarded and the medium was changed. The cells were passaged when the total population reached 70% confluence. The BMMSCs that has been passaged for 3 generations were used for injecting into the SCID mice. Additionally, 10 SCID mice were randomly divided into 2 groups with 5 mice in each group. In the control group, each mouse was subcutaneously injected 1 × 10^6^/200 μlRM-1 while the mouse in the treatment group were subcutaneously injected 1 × 10^6^/200 μlRM-1+BMSCs (9:1). Then, the tumour volumes were measured at one week following the injection. The mice were finally sacrificed after 28 days, and tumours were also weighed then.

### SUPPLEMENTARY MATERIALS FIGURES


