# Supplementary material for: Bone marrow mesenchymal stem cells participate in prostate carcinogenesis and promote growth of prostate cancer by cell fusion in vivo
Source: Oncotarget. 2016 Apr 27;7(21):30924–34. doi: 10.18632/oncotarget.9045 (PMC5058728; doi:10.18632/oncotarget.9045)
Supplement: Supplementary file 1 [file oncotarget-07-30924-s001.pdf]

Bone marrow mesenchymal stem cells participate in prostate carcinogenesis and promote growth of prostate cancer by cell fusion *in vivo*

Supplementary Materials

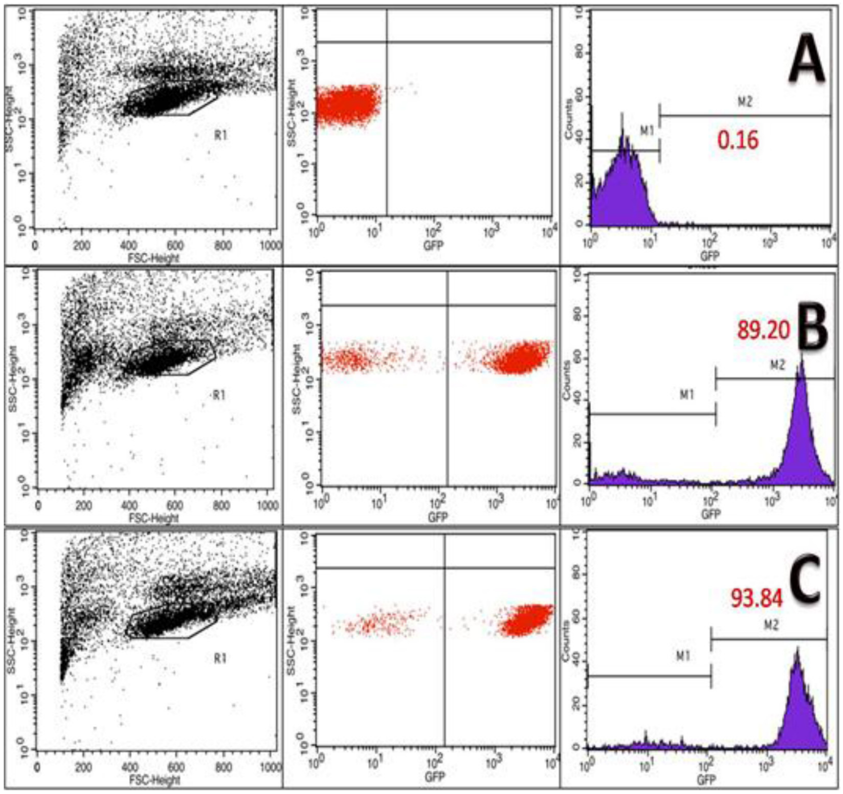

Supplementary Figure S1: The flow cytometry analysis of the peripheral blood of mice. After BMT at 4 weeks. (**A**) GFP-positive rate of peripheral blood in mice that received C57BL/6 bone marrow. (**B**, **C**) GFP-positive rate of peripheral blood in mice that received GFP bone marrow.

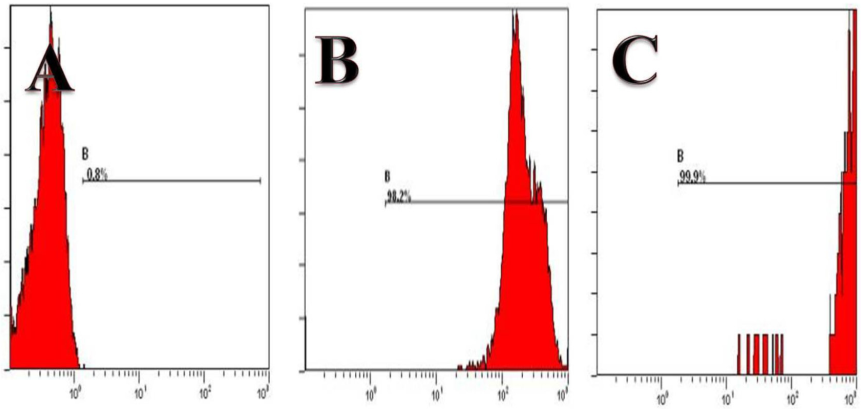

Supplementary Figure S2: The flow cytometry analysis of the bone marrow cells of mice after BMT at 28 weeks. (**A**) GFP-positive rate of bone marrow cells in mice that received C57BL/6 bone marrow. (**B**, **C**) GFP-positive rate of bone marrow cells in mice that received GFP bone marrow.

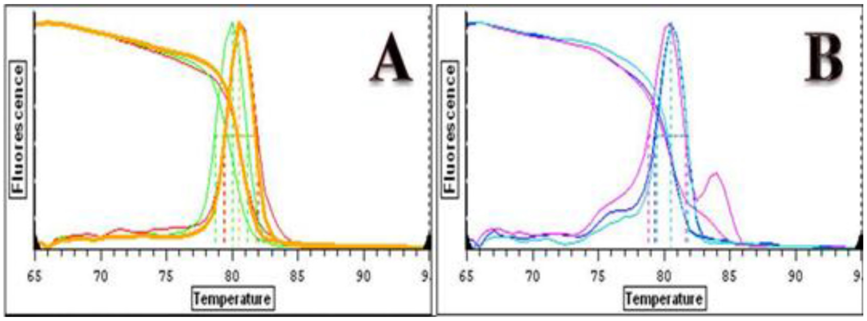

Supplementary Figure S3: The qPCR analysis for DNA of TRAMP mice. (**A**) tcrd sequence as control (**B**) SV40 sequence.

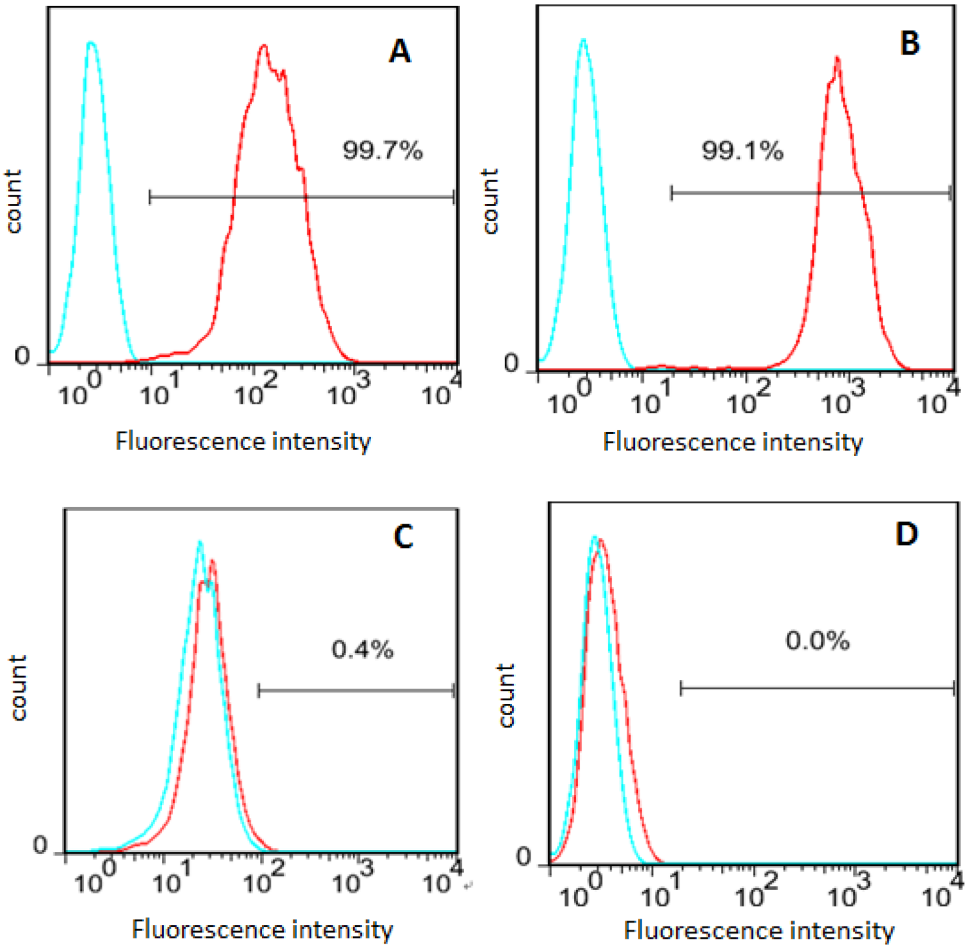

Supplementary Figure S4: Identification of the phenotype of BMMSCs. (**A**) Positive for expression of CD90. (**B**) Positive for expression of CD105. (**C**) Negative for expression of CD34. (**D**) Negative for expression of CD45 (blue lines indicate the negative control). The expression of surface markers CD90 and CD105 in BMMSCs are both positive. However, in the HSC, the expression of CD34 and CD45 are also both positive. Based on the above result, we are able to confirm the phenotype of the cells to be BMMSCs.

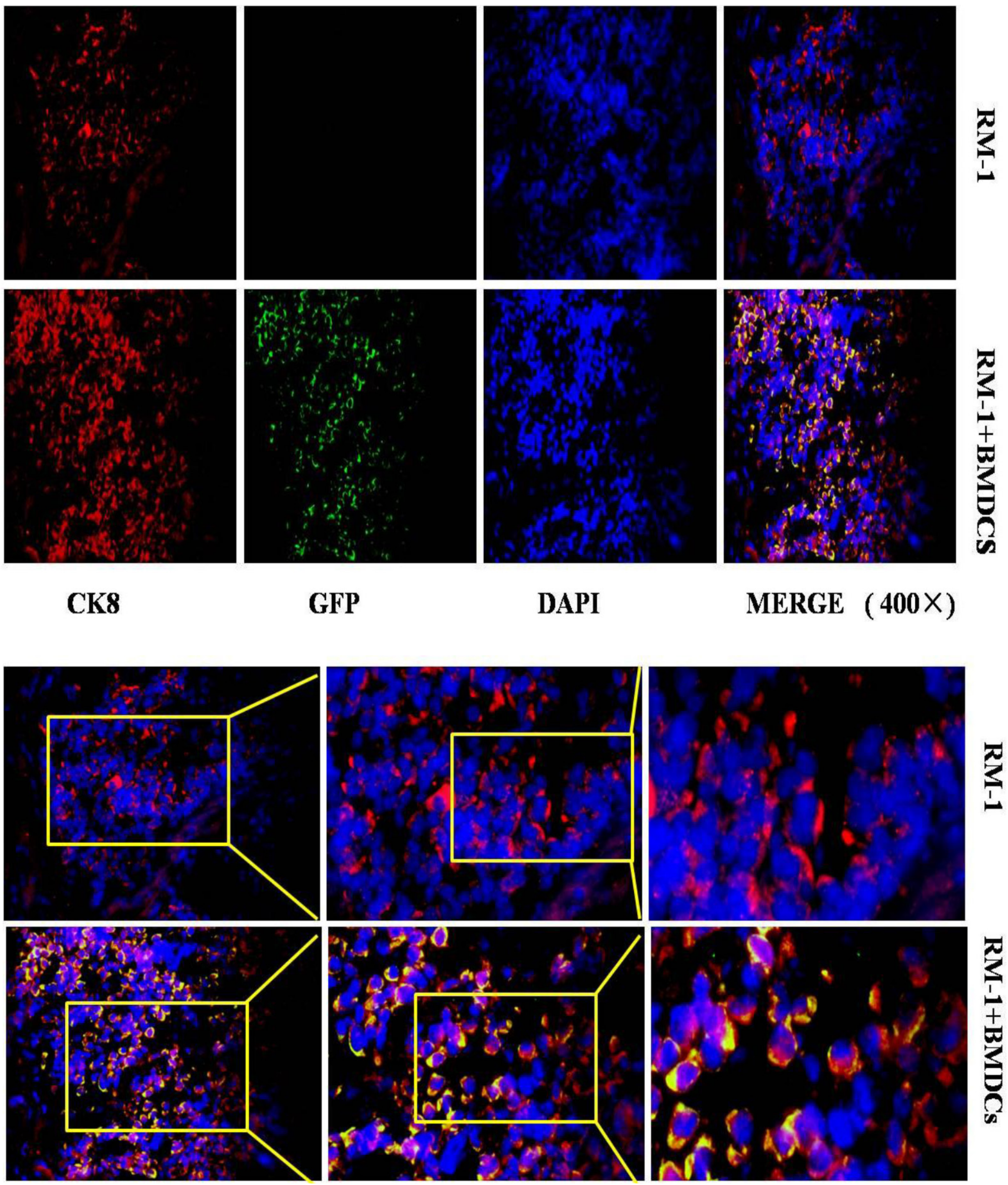

Supplementary Figure S5: GFP(+)/CK8(+) cells observed in RM-1+BMMSCs xenografts suggests that cell fusion may play a role in PCa tumor growth. The expression of CK8 and GFP in the xenografts (top panel) and cell fusion was observed in the RM-1+BMMSCs group (bottom panel).
